# First Shark from the Late Devonian (Frasnian) Gogo Formation, Western Australia Sheds New Light on the Development of Tessellated Calcified Cartilage

**DOI:** 10.1371/journal.pone.0126066

**Published:** 2015-05-28

**Authors:** John A. Long, Carole J. Burrow, Michal Ginter, John G. Maisey, Kate M. Trinajstic, Michael I. Coates, Gavin C. Young, Tim J. Senden

**Affiliations:** 1 School of Biological Sciences, Flinders University, Adelaide, Australia; 2 Department of Earth and Marine Sciences, The Australian National University, Canberra, Australian Capital Territory, Australia; 3 Geosciences, Museum Victoria, Melbourne, Victoria, Australia; 4 Ancient Environments, Queensland Museum, Hendra, Queensland, Australia; 5 Palaeontology Section, University of Warsaw, Warsaw, Poland; 6 American Museum of Natural History, New York, New York, United States of America; 7 Environment and Agriculture, Curtin University, Perth, Western Australia, Australia; 8 Earth and Planetary Sciences, Western Australian Museum, Perth, Western Australia, Australia; 9 Department of Organismal Biology and Anatomy, University of Chicago, Chicago, Illinois, United States of America; 10 Department of Applied Mathematics, The Australian National University, Canberra, Australian Capital Territory, Australia; Raymond M. Alf Museum of Paleontology, UNITED STATES

## Abstract

**Background:**

Living gnathostomes (jawed vertebrates) comprise two divisions, Chondrichthyes (cartilaginous fishes, including euchondrichthyans with prismatic calcified cartilage, and extinct stem chondrichthyans) and Osteichthyes (bony fishes including tetrapods). Most of the early chondrichthyan (‘shark’) record is based upon isolated teeth, spines, and scales, with the oldest articulated sharks that exhibit major diagnostic characters of the group—prismatic calcified cartilage and pelvic claspers in males—being from the latest Devonian, c. 360 Mya. This paucity of information about early chondrichthyan anatomy is mainly due to their lack of endoskeletal bone and consequent low preservation potential.

**Methodology/Principal Findings:**

Here we present new data from the first well-preserved chondrichthyan fossil from the early Late Devonian (ca. 380–384 Mya) Gogo Formation Lägerstatte of Western Australia. The specimen is the first Devonian shark body fossil to be acid-prepared, revealing the endoskeletal elements as three-dimensional undistorted units: Meckel’s cartilages, nasal, ceratohyal, basibranchial and possible epibranchial cartilages, plus left and right scapulocoracoids, as well as teeth and scales. This unique specimen is assigned to *Gogoselachus lynnbeazleyae* n. gen. n. sp.

**Conclusions/Significance:**

The Meckel’s cartilages show a jaw articulation surface dominated by an expansive cotylus, and a small mandibular knob, an unusual condition for chondrichthyans. The scapulocoracoid of the new specimen shows evidence of two pectoral fin basal articulation facets, differing from the standard condition for early gnathostomes which have either one or three articulations. The tooth structure is intermediate between the ‘primitive’ ctenacanthiform and symmoriiform condition, and more derived forms with a euselachian-type base. Of special interest is the highly distinctive type of calcified cartilage forming the endoskeleton, comprising multiple layers of nonprismatic subpolygonal tesserae separated by a cellular matrix, interpreted as a transitional step toward the tessellated prismatic calcified cartilage that is recognized as the main diagnostic character of the chondrichthyans.

## Introduction

Relationships between early gnathostome groups have long been a contentious issue [1, 2]. Renewed interest and debate [3–7] have been prompted by discoveries that are transforming ideas about the diagnostic characteristics of the major clades. Eye stalks, long thought to be unique to placoderms and chondrichthyans, are now known in primitive osteichthyans [8,9]. Neurocranial fissures, considered synapomorphies of acanthodians and osteichthyans [2,10,11] are now recognized in mid-Palaeozoic chondrichthyans [12–14]. Paired fin spines, once thought restricted to acanthodians, are now recognized in stem chondrichthyans [15] and osteichthyans [16–17]. Endoskeletal bone, the absence of which is commonly used to characterize chondrichthyans, is now known in some fossil [18] and living sharks [19,20]. Gill-arches having an osteichthyan-like pattern are now also known in certain early shark fossils [21]. Despite all these new data, the paucity of well-preserved chondrichthyan fossils (in the traditional or conventional sense of the group, excluding ‘acanthodian’ genera now linked to the chondrichthyan stem lineage [3–7]) which are older than Late Devonian in age [22–24] has greatly hampered the resolution of deep gnathostome phylogeny.

In recent years, well-preserved discoveries of Devonian fossils have provided new data on the morphology of early chondrichthyans. Such discoveries include the first articulated specimen of *Doliodus problematicus* from the Early Devonian of Canada [15, 25] and isolated neurocrania of Early–Middle Devonian sharks from South America [13, 26] and Germany [27], plus an articulated neurocranium and visceral skeleton from South Africa [28] which have produced a wealth of anatomical information through CT scanning. The acid-prepared specimen we describe here was preserved in a limestone concretion from the Late Devonian (early Frasnian) Gogo Formation, Western Australia. The fishes of the Gogo Formation are well known for their exceptional 3-D preservation not only of skeletal material [29–35] but also of soft tissues including muscles [36, 37], embryos and maternal feeding structures [38,39]. Their anatomical data has contributed greatly to resolving problems of osteichthyan and placoderm phylogeny [2–7,28–31,34, 36,37,40]. The fauna includes a high diversity (c. 50 species) of placoderms and osteichthyans, and a single acanthodian [41], but no previous chondrichthyan specimens had been discovered during more than 60 years of collecting at the site [42].

The fossils occur inside limestone concretions which formed around whole fish or parts of carcasses that fell into the deep inter-reef basins between the reef fronts. Rapid calcitic concretion formation prevented compaction or distortion from tectonic or gravity-induced sedimentary loading, thus retaining the original shape of the bones and delicate perichondrally calcified cartilages [34]. Geochemical studies indicate that carcasses fell through a euxinic layer within the basin, enabling rare rapid preservation of soft tissue in anoxic benthic conditions [43].

**Fig 1:**
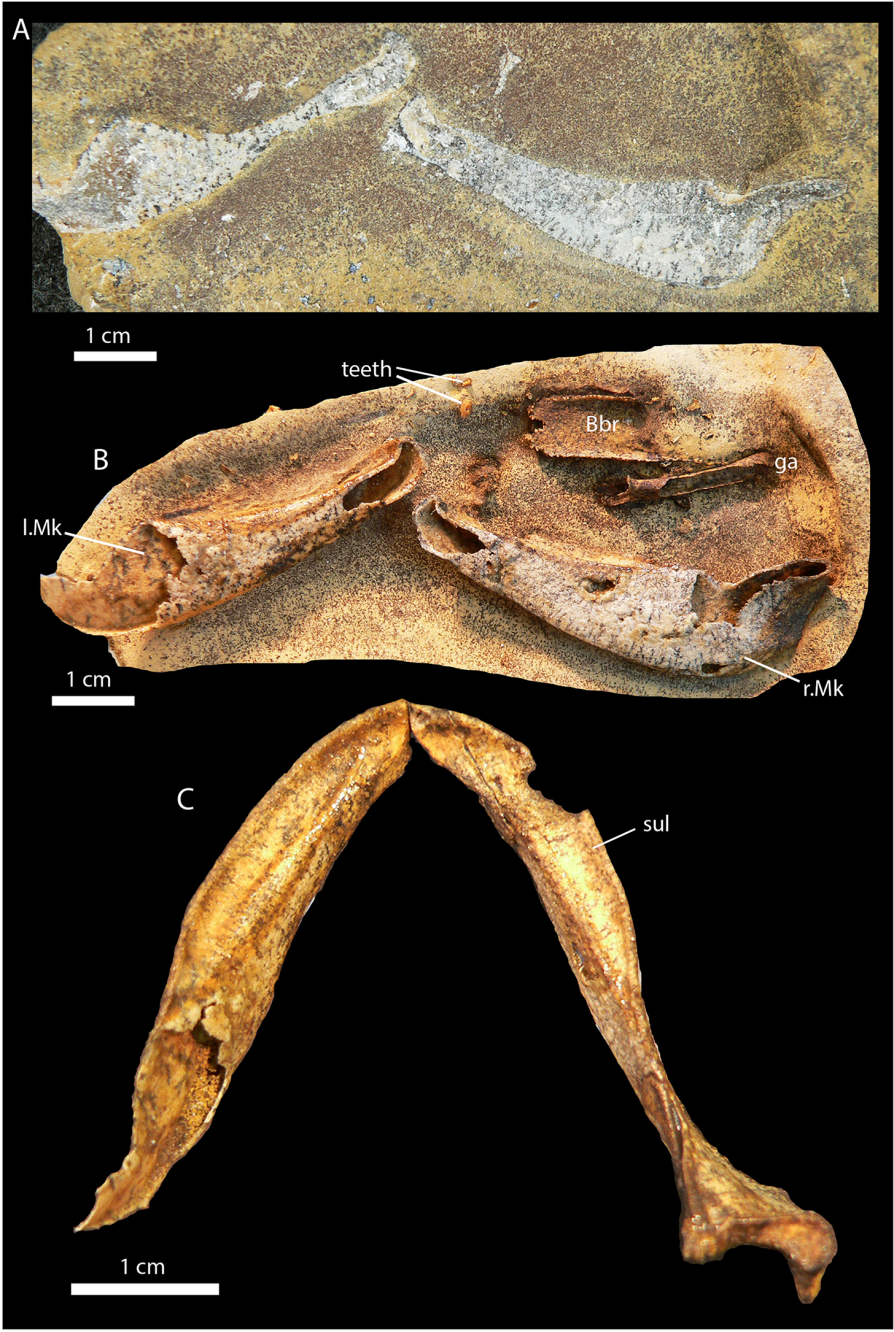
Preparation of *Gogoselachus lynbeazleyae* WAM 09.6.145, Gogo Formation, Western Australia. (A) Meckel's cartilage as exposed on collection, before acetic acid etching. (B) specimen during early acid preparation. (C) Meckel's cartilages WAM 09.6.145–001 (left), WAM 09.6.145–002 (right) after full preparation, shown articulated in dorsal view.

*Gogoselachus lynbeazleyae* is represented by a set of associated elements which were probably held together by ligaments and muscular tissue when the concretion formed around them. They include both left and right Meckel's cartilages, nasal cartilage, ceratohyal, hyomandibula, basibranchial cartilage, both scapulocoracoids, as well as associated teeth and scales. Fig 1 shows the specimen as it was found in the field (Fig 1A), after a week in acetic acid (Fig 1B), and after the lower jaws had been freed and reassembled into life position (Fig 1C).

Very few chondrichthyan remains of this age are preserved in 3D form, and those which have been visualized in 3D are mostly from imaged micro CT tomography [12,21,25] or from cast impressions (e.g. ‘the Tennessee cladodont’) [44]. The Gogo specimen, to our knowledge, is the first ever example of a Devonian shark in which well-mineralized skeletal remains have been acid prepared out of the matrix. Study of the new shark specimen thus provides a unique opportunity to observe the undistorted, three-dimensional shape of the individual endoskeletal elements preserved and the internal structure of the cartilage forming these elements, as well as of multiple tooth and scale variants.

## Materials and Methods

### Field Work

The field work was done in Australia with permission of the land owners and leaseholders. There are no regulations pertaining to collecting of fossils in this region of Australia apart from land owner permission as stated under the Lands Administration Act, as relevant to pastoral leaseholders: see http://www.comlaw.gov.au/Details/C2010Q00239.

The specimen is registered in the collections of The Western Australian Museum as WAM 09.6.145, repository at 69 Kew St, Welshpool, Western Australia.

### Preparation and Imaging

The specimen was acid prepared at Museum Victoria, Melbourne by JAL using 10% acetic acid, with cartilage elements strengthened by Mowital B30 in ethanol. The specimen and residues were washed in water to neutralize the acidity, then picked under a binocular microscope to retrieve isolated scales and teeth.

Scanning electron micrographs of teeth, scales and cartilage structure were taken using a JEOL-6400 at the Centre for Microscopy, University of Queensland (scales), and using other SEMs at University of Warsaw, Poland (teeth images), and the Los Angeles County Museum of Natural History, California (cartilage). Thin section images were taken using an Olympus BX-50 microscope with DP12 imaging system.

Digital transverse sections of the cartilages were studied through composite serial reconstruction from 10μ slices by the ultrafine CT scanner in the Dept of Applied Mathematics, ANU, and studied using Drishti 2.4 software developed by that department (and now publically available as freeware).

### Nomenclatural Acts

The electronic edition of this article conforms to the requirements of the amended International Code of Zoological Nomenclature, and hence the new names contained herein are available under that Code from the electronic edition of this article. This published work and the nomenclatural acts it contains have been registered in ZooBank, the online registration system for the ICZN. The ZooBank LSIDs (Life Science Identifiers) can be resolved and the associated information viewed through any standard web browser by appending the LSID to the prefix "http://zoobank.org/". The LSID for this publication is: urn:lsid:zoobank.org:pub: 3563562E-41E0-4579-8645-9C13F7880019. The electronic edition of this work was published in a journal with an ISSN, and has been archived and is available from the following digital repositories: PubMed Central, LOCKSS.

### Institutional Abbreviations

**AEU,** Department of geology, Azad University, Esfahan, Iran.

**QM,** Queensland Museum, PO Box 3300, South Brisbane BC, Queensland 4101.

Fossil collections located at Hendra Annex.

**WAM,** Western Australian Museum Fossil Collection; 49 Kew St, Welshpool, WA, 6106.

## Results

### Systematic Palaeontology

Chondrichthyes Huxley, 1880

Order, family indet.

***Gogoselachus lynbeazelyae*** gen. et sp.nov. Long, Burrow, Ginter, Maisey, Trinajstic, Coates, Young, Senden urn:lsid:zoobank.org:act:EF04AD3E-865D-4FA5-A6FF-F4ED6359FD26; urn:lsid:zoobank.org:act:2ED167F8-9506-4D43-B08E-237291937B11

#### Etymology

Generic name for the Gogo Formation and Greek ‘*selachos*’, shark. Species name acknowledging Professor Lyn Beazley, of the University of Western Australia, for her contribution to scientific progress in Western Australia.

#### Holotype

WAM 09.6.145.

#### Type species

*Gogoselachus lynbeazleyae* sp.nov.

#### Synonymy

? *Deihim mansureae* "dubious specimen AEU 236" Ginter et al. (2002; plate 4, figures J–K) [45]. "shark" Long & Trinajstic (2010; p.263, figure 2) [34]

#### Type Locality

Circa 60 km SE of Fitzroy Crossing, north Western Australia, near the Stromatoporoid Camp locality, collected by JAL, July 7th, 2005.

#### Horizon & Age

Late Devonian (early Frasnian) Gogo Formation, Canning Basin.

#### Diagnosis

A chondrichthyan with cladodont type teeth characterised by the following combination of features: a crown with a prominent median cusp rounded in cross-section, two smaller lateral cusps, and, in asymmetrical forms, a single intermediate lateral cusplet situated between the median cusp and the mesial lateral cusp; a row of slender accessory labial cusplets at the crown-base interface; a euselachian type base with a deep aboral depression and a vague orolingual hump. Meckel’s cartilage with wide, transverse articular cotylus and small mandibular knob; ceratohyal with a deep anterior-facing pit towards the posterior of the outer surface; elongate basibranchial with straight edges; scapulocoracoid with two diazonal nerve foramina and two pectoral fin basal articulation facets.

## Description

### Endoskeleton

**Fig 2:**
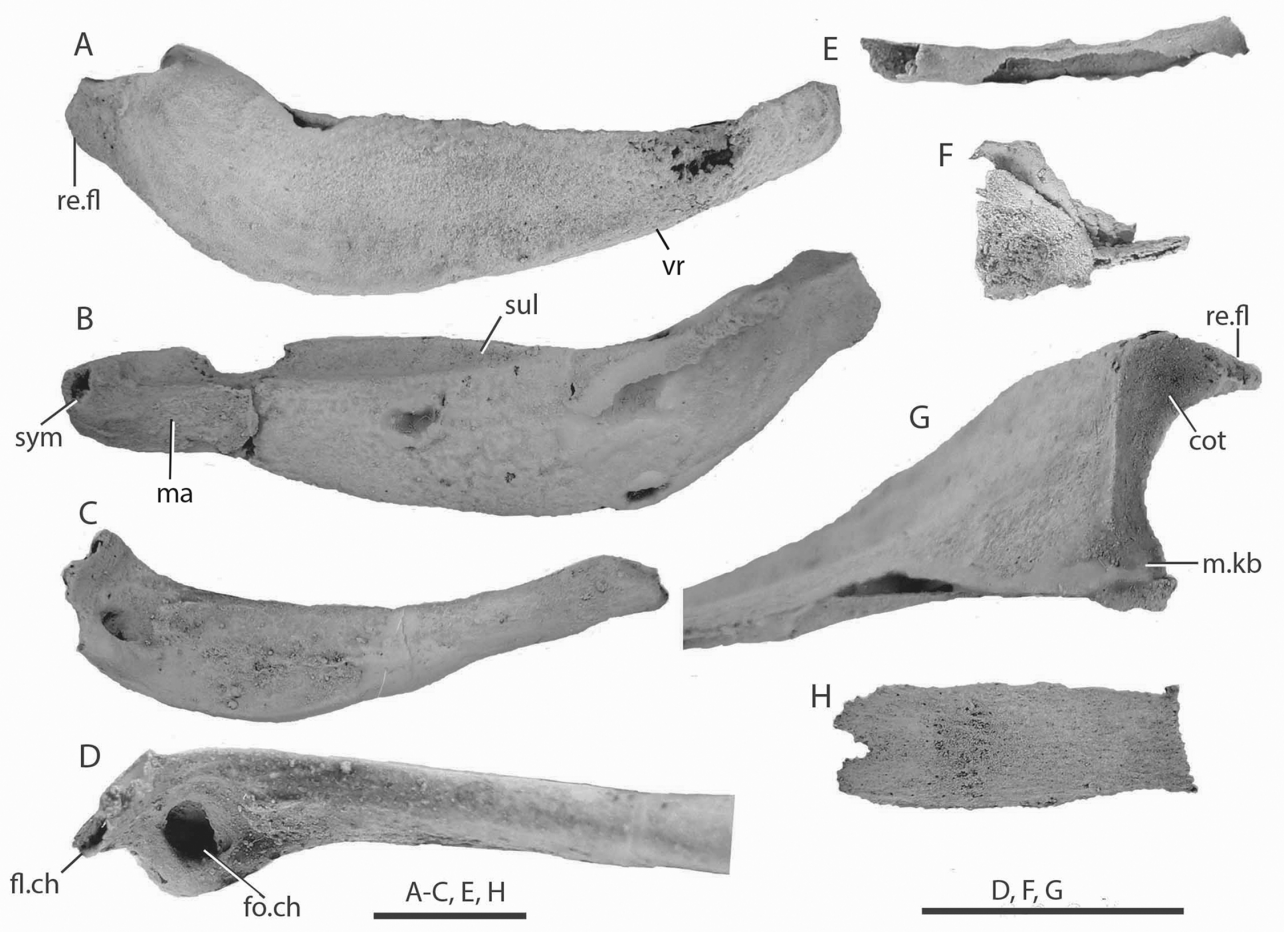
Head and branchial cartilages of *Gogoselachus lynbeazleyae* WAM 09.6.145, Gogo Formation, Western Australia. (A–C) left Meckel’s cartilage WAM 09.6.145–001, medial, lateral and dorsal views. (D, E) ceratohyal WAM 09.6.145–005, lateral and dorsal views. (F) nasal cartilage WAM 09.6.145–006, anterior vie w?. (G)? epibranchial cartilage WAM 09.6.145–004. (H) basibranchial cartilage WAM 09.6.145–003. Abbreviations: cot, cotylus; fl.ch, flange on ceratohyal; fo.ch, fossa on ceratohyal; ma, muscle attachment area; m.kb, mandibular knob; re.fl, retroarticular flange; sul, sulcus; sym, symphyseal pit; vr, ventral ridge.

The Meckel’s cartilages (Figs 1A–1C; 2A, 2B and 2G) are c. 5 cm long, deep posteriorly, with a well-developed continuous dental sulcus for the tooth rows (sul), bordered laterally by a raised straight rim. The outer face of the cartilage has a ventral ridge (Fig 1A, vr) defining a depressed lateral surface for the insertion of the adductor mandibulae musculature. The lower jaw articulatory surface has a broad transverse cotylus (Fig 2G, cot) flanked medially by a small mandibular knob (m.kb). Both the cotylus and the mandibular process are on the same transverse plane. Posterior to the cotylus is a thin vertical lamina (Fig 2A and 2C, re.fl), the sustentaculum *sensu* Gegenbaur [46] a structure that has been recently referred to as the retroarticular flange [22]. The anterior region of the Meckel’s cartilage has a well-defined oval symphysial attachment area (Fig 2B, sym) with a separate subrectangular muscle attachment area posteroventral to the symphysis (ma).

The ceratohyal (Fig 2C and 2D) conforms to the generalized chondrichthyan shape [44,46], having an expanded posterior blade and narrow, slightly rotated anterior region. It is about three-quarters the length of the Meckel’s cartilage, and has a large fossa (fo.ch) facing anteriorly at its posterior extremity, rimmed by an elevated flange (fl.ch). The anterior end of the ceratohyal is slightly expanded, with two separate, slightly rugose areas on the medial surface. It bears no distinctive grooves or foramina.

A delicate, triangular double-lamina of twisted cartilage resembles a nasal cartilage (Fig 2F). It correlates closely to the size and shape of other primitive chondrichthyan nasal cartilages or parts of the nasal capsule [14]. A shorter, tubular cartilage preserved above the Meckel’s cartilages is one of the gill arches (Fig 2E), most likely epibranchial 1 by comparison with *Ozarcus* [21]. A single elongate and transversely concave cartilage plate probably represents the mineralized surface of a basibranchial cartilage (Fig 2H). It has a straight posterior margin with dorsally projecting corners; the anterior end of the unit is M-shaped. Midway along, its convex surface is rough with numerous small pores.

**Fig 3:**
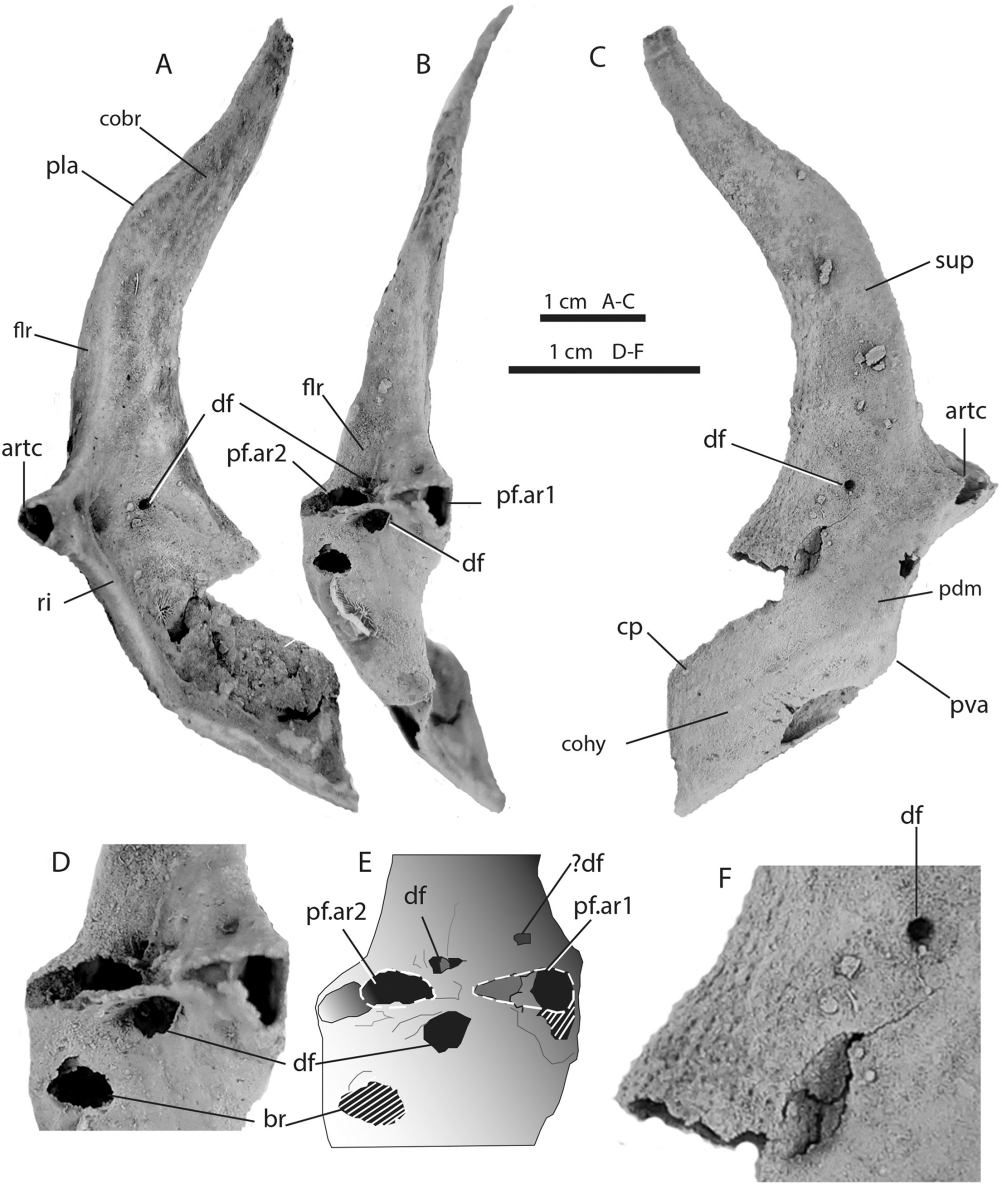
Left scapulocoracoid WAM 09.6.145–007 of *Gogoselachus lynbeazleyae*, Gogo Formation, Western Australia. (A) posterior (B) medial and (C) lateral views. D, close up of posterior face showing articulation area for pectoral fin, E., interpretation of same area. F, close up of central lateral surface showing diazonal foramen (df). Abbreviations: af, articulation facets; artc, articular crest; br, break in bone; cobr, coracobrachialis muscle attachment area; cohy, coracohyoideus muscle attachment area; cp, coracoid plate; df, diazonal foramina; muscle attachment areas; flr, mediolateral pectoral retractor muscle attachment area; pdm, pectoral depressor muscle attachment area; pla, posterolateral process; pf.ar1,2, pectoral fin articulation areas 1 and 2; pla, posterolateral angle; pva, posteroventral angle; ri, ridge; sup, supinator attachment area.

**Fig 4:**
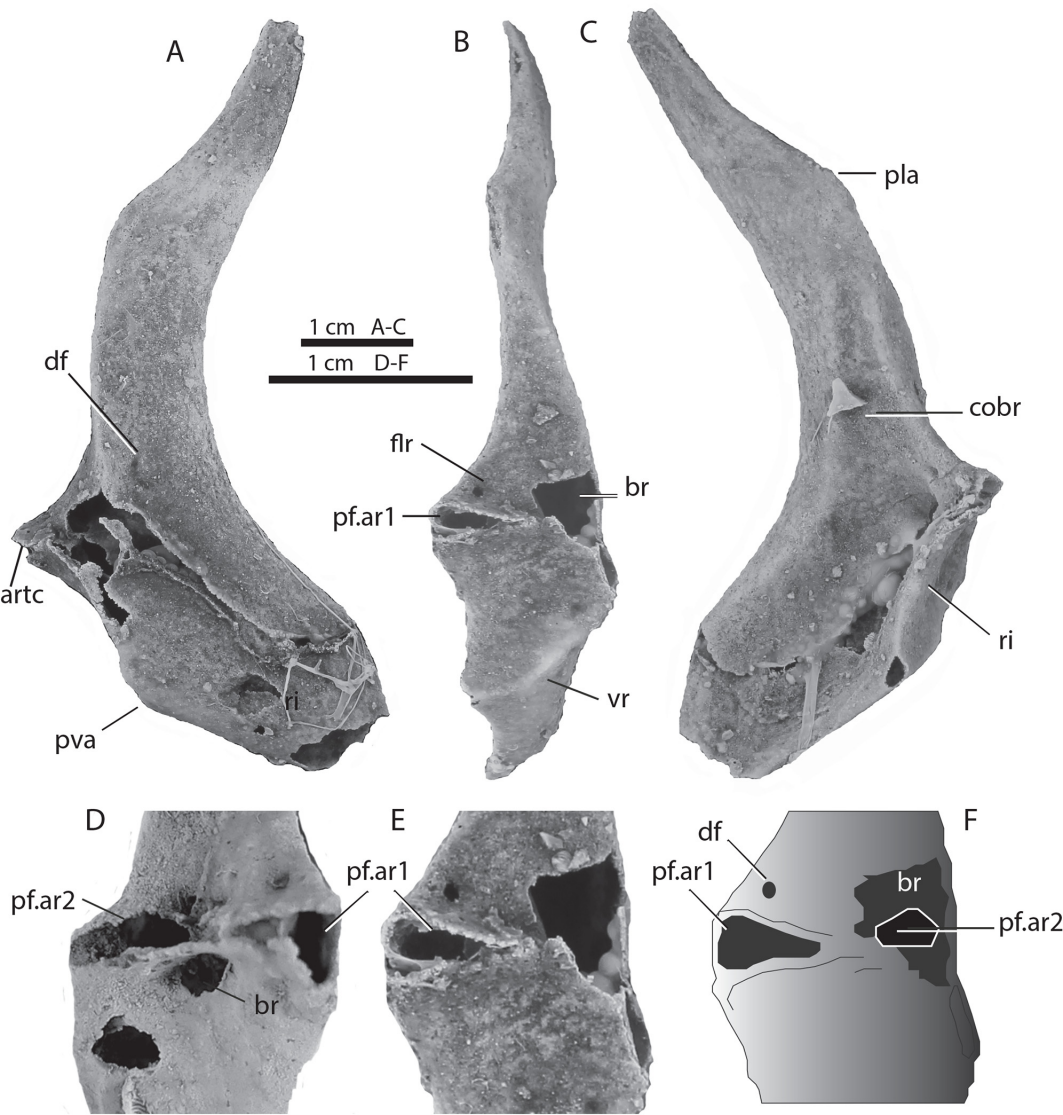
Right scapulocoracoid WAM 09.6.145–007 of *Gogoselachus lynbeazleyae*, Gogo Formation, Western Australia. (A) lateral (B) posterior and (C) medial views, with close up of articulatory area for pectoral fin in E, F. D, articulation area for pectoral fin articulation on left scapulocoracoid for comparison. Abbreviations as for Fig 3.

The scapulocoracoid (Figs 3 and 4) is 8.0 cm high, with an elongate, flat, dorsally tapering scapular region and a deeply concave coracoid region ventral to a thickened posterior ridge at the lateral inflection. The scapular blade has a weak posterolateral process (pla). Muscle attachment scars are interpreted as follows: the supinator muscle anteriorly on the lateral surface of the scapula (sup); the mediolateral pectoral retractor muscles near the anterolateral inflection (flr); the coracobranchialis muscles (cobr) above the inflection ridge; the coracohyoideus muscles (cohy) ventrally; and the pectoral depressor muscle sheet (pdm) on the posterolateral surface of the coracoid (Fig 3A and 3B). A ridge (ri) along the posterior angle of the ventrolateral inflection shows remnants of areas with raised borders enclosing surfaces presumed to be articulation facets for the pectoral fin basal cartilages. The small central facet is completely preserved on the left side (Fig 3B and 3D, pf.ar2), but the lateral and medial surfaces of the ridge have broken off. The medial area shows remnants of a rim shared with the central area, but the lateral area is missing.

The left scapulocoracoid is slightly damaged in the area of the fin articulation but clearly shows two facets for fin radial articulations (Fig 3D and 3E). This interpretation is supported by features preserved in the right scapulocoracoid which has a broad triangular facet for the mesial pectoral fin radial attachment (Fig 4B and 4E, pf.ar1) clearly visible, as well as a slightly smaller lateral pectoral fin radial facet (pf.ar2). Restorations combining data from both sides show that there was only two fin radial articulations (Fig 3E,Fig 4F).

### Teeth

**Fig 5:**
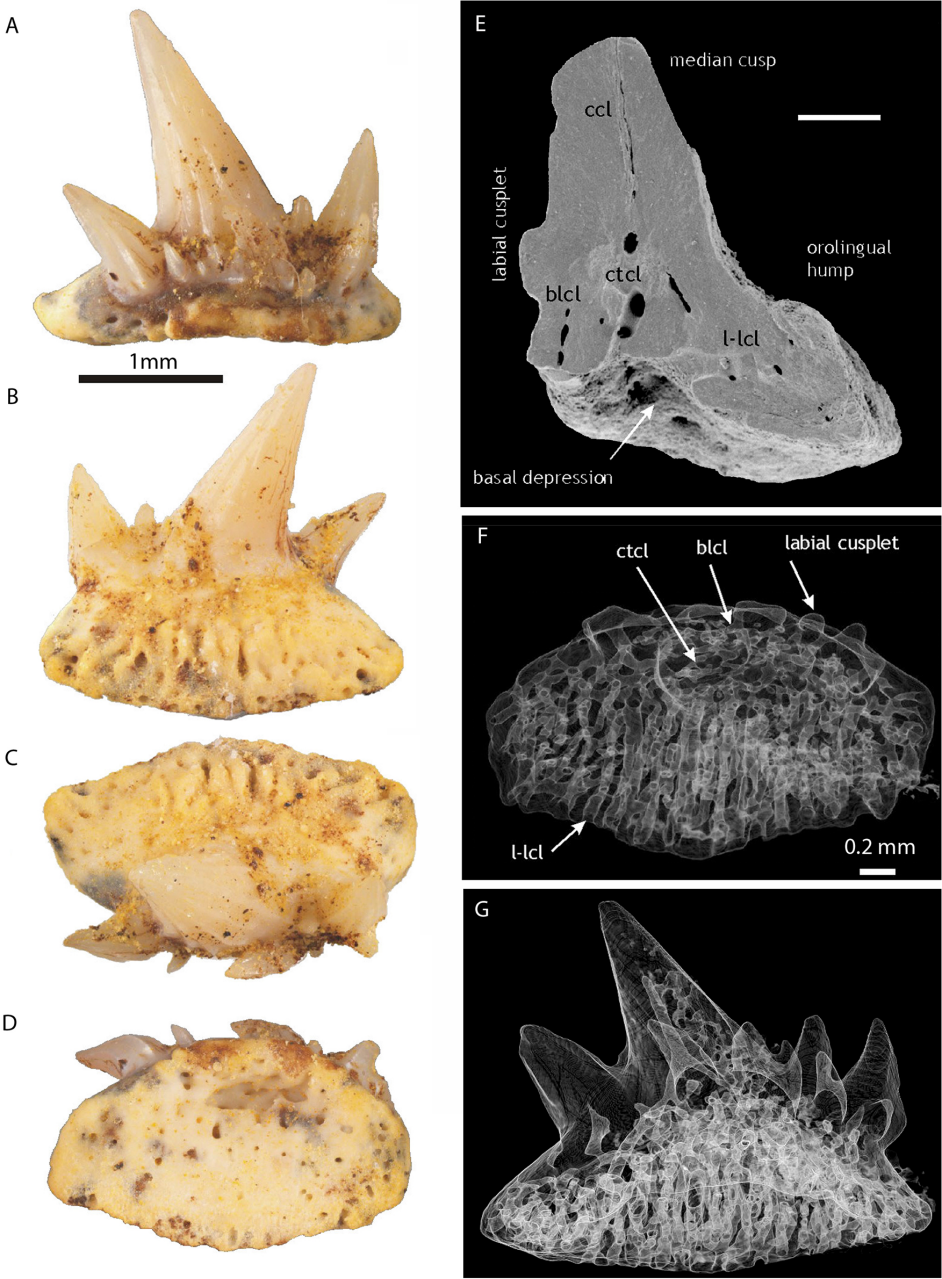
Teeth of *Gogoselachus lynbeazleyae* WAM 09.6.145, Gogo Formation, Western Australia. Large tooth WAM 09.6.145–009 in (A) lingual, (B) labial, (C) oral (D) aboral views, in natural light. (E) SEM of tooth WAM 09.6.145–010, naturally broken near the median surface. (F, G) CT-scans of tooth WAM 09.6.145–011, showing layout of vascular canals, in basal and lingual-lateral views. Abbreviations: l-lcl, labio-lingual canals; ctcl, central canal; blcl,basolabial canals; ccl, coronal canals.

**Fig 6:**
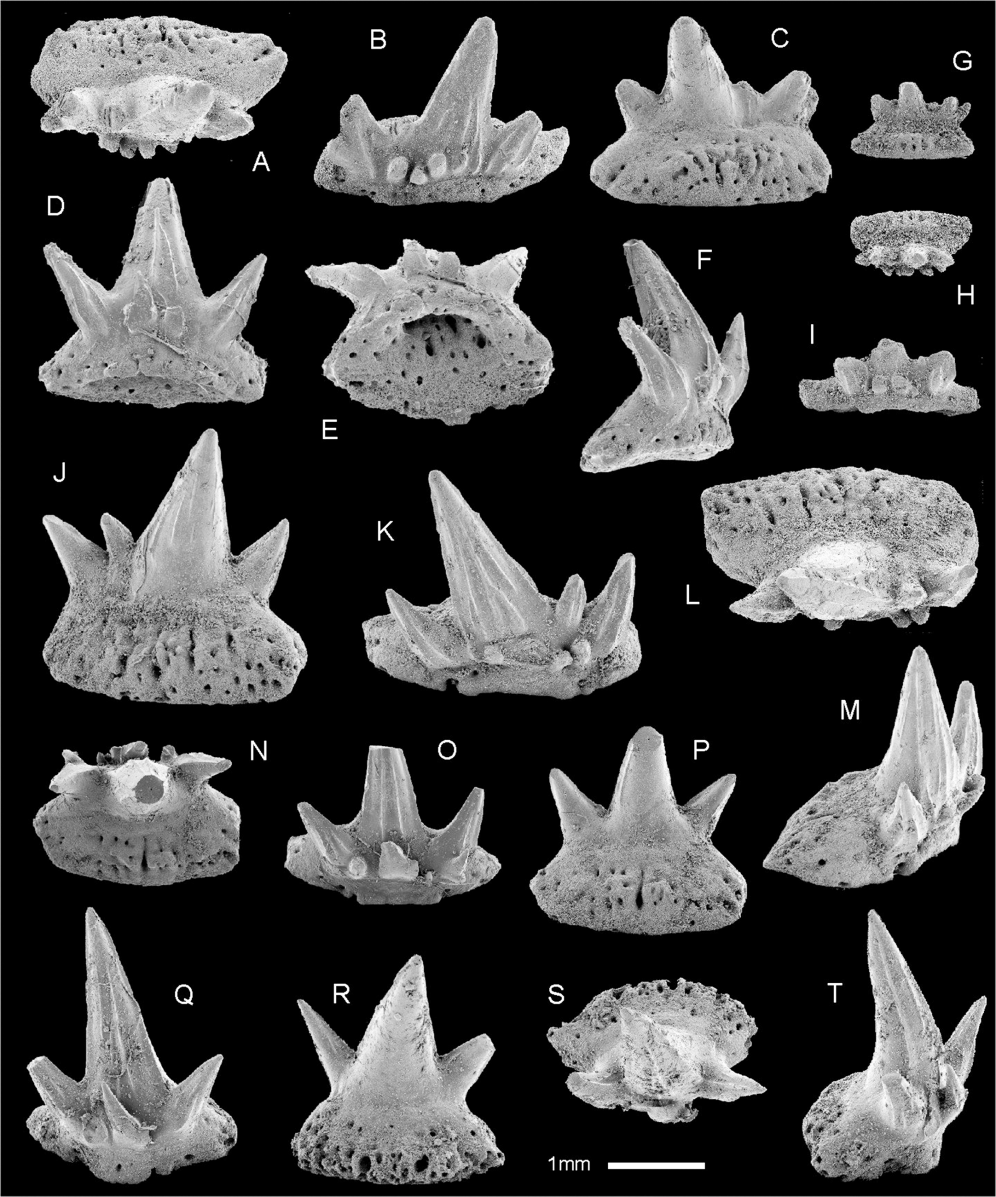
SEM images of teeth of *Gogoselachus lynbeazleyae* WAM 09.6.145, Gogo Formation, Western Australia. (A–C) WAM 09.6.145–012 in oral, labial, and lingual views. (D–F) WAM 09.6.145–013 in labial, basal, and oblique lateral views. (G–H) Small tooth, WAM 09.6.145–014, in lingual, and oral views. (I) Tooth of intermediate size, WAM 09.6.145–015, in labial view. (J–M) WAM 09.6.145–016 in lingual, labial, oral and distal views. (N–P) WAM 09.6.145–017 in oral, labial, and lingual views. (Q–T) WAM 09.6.145–018 in labial, lingual, oral, and lateral views.

The dentition is represented by some 82 individual cladodont-type teeth which show limited morphological variation; more than half are relatively large (width at the base–crown junction 1.5–2 mm; Figs 5A–5E; 6A–6F and 6J–6T) with the others smaller (all about 1 mm wide; Fig 6G–6I). Two types of crowns, symmetrical and asymmetrical, were observed among the larger teeth. Symmetrical crowns (Fig 6D–6F and 6N–6T) have a prominent median cusp, standing upright (in labial view) or with a slight distal inclination, and only two divergent lateral cusps whose height usually exceeds one-third of the height of the median cusp. No intermediate cusplets are present. In asymmetrical crowns (Figs 5A–5D; 6A–6C, 6J–6M) the median cusp is considerably inclined distally (up to 20 degrees), the distal lateral cusp is inclined distally at 45 degrees and the mesial lateral cusp is only slightly inclined mesially. Between the median and mesial cusps there is a small intermediate cusplet. The asymmetrical teeth are wider (mesio-distally) than the symmetrical ones. The median cusp is up to 2.5 mm in height in symmetrical teeth; in asymmetrical teeth it is relatively shorter and thicker. In all types of teeth it is rounded in cross section, gently curved lingually, but non-sigmoidal. The labial face is ornamented with two to three coarse cristae almost reaching the tip; the cristae may bifurcate at the basal part. The lingual face is almost smooth, only one or two indistinct cristae extend along the lateral margins. In asymmetrical specimens they are visible near the mesial margin (Fig 6C and 6J). The lingual and labial faces are separated with a lateral carina. The shape and ornamentation of the lateral cusps is similar to that of the median cusp.

At the crown–base interface, on the labial side of all the teeth, is a row of several accessory cusplets. They display various sizes and directions, but generally are slender, sharp and directed orally. In the asymmetrical specimens the largest may exceed the size of the intermediate cusplet. The labial accessory cusplets are vulnerable to abrasion and in most cases they are broken, leaving only the basal parts preserved.

Tooth bases of larger teeth are deep, elongated mesio-distally, from elliptical to broadly hexagonal, to trapezoidal in outline, with a lingual extension. A deep depression in the central/labial area of the aboral side is framed by the well developed, labially convex basolabial rim. No clear interlocking devices (buttons or basolabial projections) are present, save for a slight orolingual hump, observed in certain, especially small, specimens. The aboral depression and the basolabial rim might have helped in strengthening the connection between the teeth in a tooth file. The base surface, save for the immediate vicinity of the crown on the oral side and the aboral-lingual area, is perforated by numerous canal openings. The largest foramina occur on the orolingual rim and in the aboral depression.

The CT-scans and broken surfaces reveal a network of basal nutritive canals (Fig 5E–5G). In the lingual extension of the base there are mainly labio-lingual canals which, in most cases, extend from the orolingual rim to the lingual wall of the aboral depression. These major canals are interconnected by narrower tubes. In the area of the depression (i.e. below the crown; called here central canals), the directions change to mainly mesio-distal and vertical. And finally, the canals in the basolabial rim are mainly vertical. From the network of central canals, single vascular canals (coronal canals) extend into the basal parts of the median and lateral cusps.

The smaller teeth (Fig 6G–6I) differ from the larger ones not only by the overall size, but also in proportions between the base and crown—in the smaller teeth, in most cases, the base is relatively broader (see especially Fig 6I). In contrast to the excellent preservation of many of the larger teeth, most of the recovered smaller teeth have broken cusps. As the teeth were not found in situ, but dispersed around the jaw cartilages, we can only speculate in reconstructing the dentition. It is most probable that the narrow, symmetrical teeth were on the anterior part of the jaw, and the broader, asymmetrical forms were in the lateral tooth families. The smaller teeth are probably juvenile ones, replaced by larger forms, but preserved on the outer side of jaw, as is common among the ctenacanthiforms [47–49]. The occurrence of both symmetrical and asymmetrical forms among the smaller teeth, as well as their broken (possibly some worn) cusps, support this view.

Tooth bases resemble the “euselachian-type” (*sensu* Ginter 2005) [50] in having a spongiose structure and numerous labio-lingual canals. The absence of buttons and basolabial projections is a potential synapomorphy with chondrichthyans possessing "euselachian-type" teeth, although the presence of an orolingual hump and basolabial depression suggests that the bases of successive teeth overlapped in similar fashion to those of cladodont chondrichthyans in which buttons and projections are present [47,49–52]. The presence of one or two medially situated larger canals in bases of symmetrical teeth (Fig 6P and 6R), instead of evenly distributed canals of virtually the same size, possibly represents a stage between the layout in ctenacanthiforms and symmoriiforms, which have median basal canal openings (lingual and aboral) which are usually much larger than any other foramina, and "euselachian-type" teeth with evenly distributed canals.

### Scales

**Fig 7:**
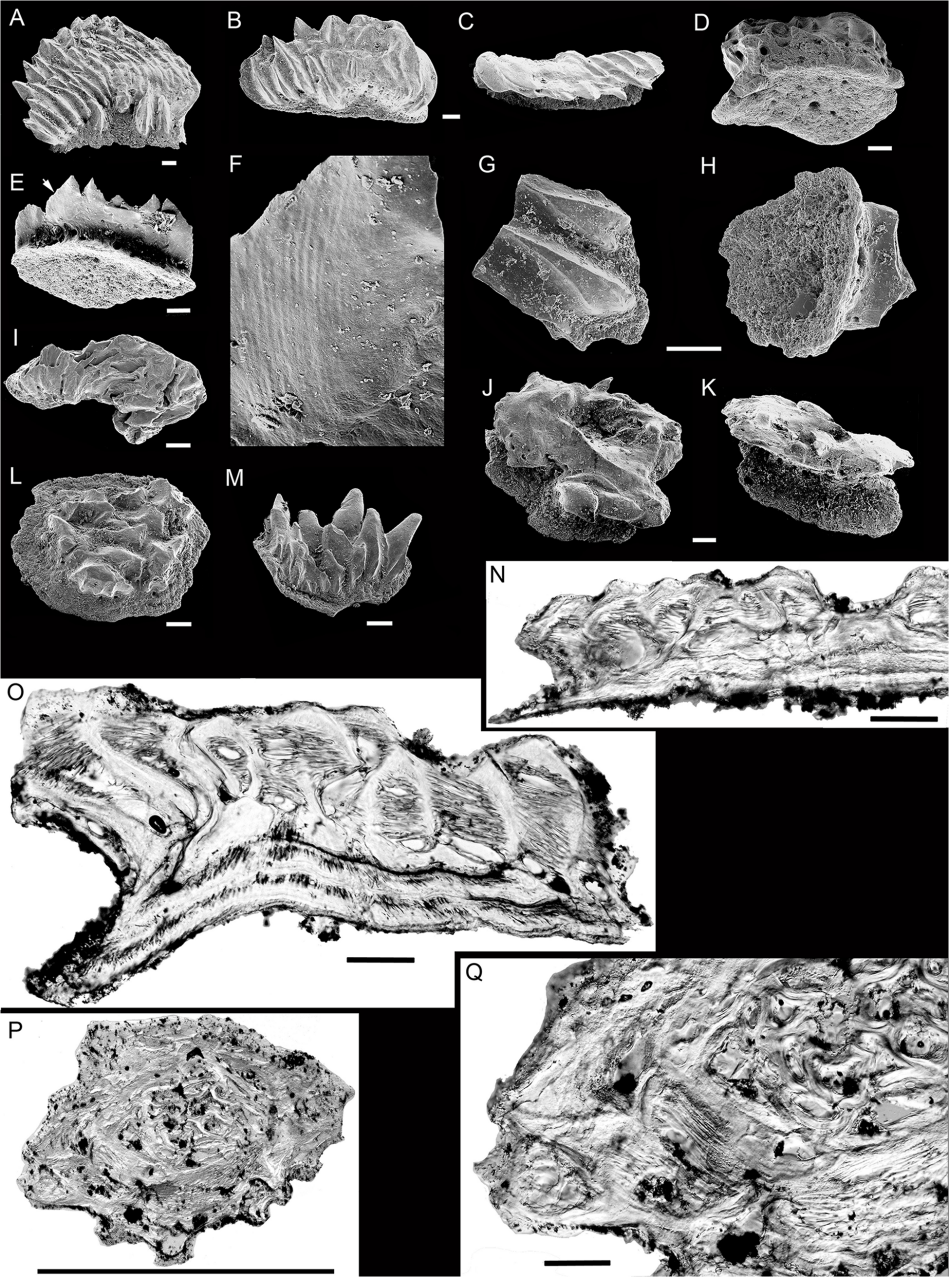
SEM images of scales of *Gogoselachus lynbeazleyae* WAM 09.6.145, Gogo Formation, Western Australia. (A) presumed flank scale WAM 09.6.145–019, anterocrown view. (B, C) presumed flank scale WAM 09.6.145–020, anterocrown and posterior views. (D) presumed flank scale WAM 09.6.145–021, posterobasal view. (E, F) presumed flank scale WAM 09.6.145–022, posterobasal view and magnification of undersurface of crown denticulation. (G, H) very small scale WAM 09.6.145–023, broken, showing crown view of one half and basal view of the other half. (I) abraded umbellate scale WAM 09.6.145–024, crown view. (J, K) Pultschuppe or squamae proniae scale WAM 09.6.145–025, crown and posterior views. (L) stellate tessera WAM 09.6.145–026, crown view. (M) probable branchial denticle plate WAM 09.6.145–027, lateral view. (N) vertical transverse section through presumed flank scale WAM 09.6.145–028. (O) oblique vertical section through umbellate scale WAM 09.6.145–029. (P, Q) horizontal section through the crown of? head tessera WAM 09.6.145–030, whole section and magnification to show Spiralfasern structure. Scale bars = 0.1 mm in all figures except (P), where scale bar = 1 mm.

Presumed body scales are of ctenacanth-type, similar to those on nearly all other articulated Devonian chondrichthyans, with a crown formed of many elongated, pointed odontodes (Fig 7A–7C, 7I, 7L and 7M). The odontodes are mostly horizontal, radiating back from a central embayment on the anterior edge. *Gogoselachus lynbeazleyae* also has other scale forms, including head tesserae, umbellate and other specialized scales (Fig 7), which have previously been described in the ctenacanthoid referred to *Tamiobatis vetustus* [22]. All scales and platelets have a thin concave base with a diamond-shaped or sub-rectangular outline, that is mostly smooth except for scattered pore openings on most scales (Fig 7D and 7E), or open pulp canals on the smallest scales (Fig 7H). The commonest forms (Fig 7A–7F), presumed to be normal body scales, have a crown formed of multiple finger-like odontodes each with a concave upper surface. The scales are mostly flat, wider than long, with their length increasing relative to width as the number of odontodes increases. A narrow margin along the upper anterior edge of the base is unornamented, and the crown extends beyond the posterior edge of the base, forming a sharply denticulated posterior crown margin (Fig 7B and 7E). Several pore openings are visible along the crown-base margin posteriorly (Fig 7D and 7E **),** and at the bases of some of the anteriormost odontodes. The smallest, flattest scales in the residues show external evidence of separate bases (Fig 7G and 7H, two halves of the same scale in crown and basal views respectively). Similarly sized scales of the commonest morphotype vary from being very flat to moderately flat (c. 0.1 mm vs. 0.2 mm high), perhaps dependent on the dorsal-ventral position on the body [52]. Fine subparallel striations are visible on the undersurface of the posterior crown denticulations (Fig 7F).

Rare scales (Fig 7I) resemble the squamae umbellatae (*sensu* Gross) [53]), umbellate scales which grew next to sensory lines in *Nostolepis striata* Pander and other acanthodians including *Ischnacanthus gracilis* and *Brochoadmones milesi*. Their crown is confined within the base outline, with closely bunched odontodes, but with an ornament-free embayment on one edge; the latter feature is characteristic of the umbellate scale form and could signify that the scale was positioned next to a sensory line pore. Another scale form, presumed to be from the head, is equivalent to Williams’ type III in *Tamiobatis vetustus* Eastman ([22] Fig 7E and 7F), and resembles the tesserae coronatae (*sensu* Gross) [53] of *N*. *striata*. These scales have bulbous tubercles clustered together with a narrow ornament-free margin extending more than halfway around the edge of the crown. Three such scales (two c. 0.7 mm diam., other c. 0.3 mm) were originally attached together, but separated on cleaning. Their individual base outlines are roughly circular except along relatively straight sectors where adjacent scales were attached (Fig 7J and 7K). Stellate scales or platelets resembling the tesserae stellatae [53] of *N*. *striata* and also *T*. *vetustus* type IV ([22] Fig 7H) have subcircular–polygonal base outlines, and crowns ornamented with interconnected ridges and tubercles (Fig 7L). Other scales resembling the 'Pultschuppen’ (*sensu* Gross) [53] of *N*. *striata*, have a relatively deep concave neck all round, with a centrifugal-growth crown: each new circular zone, formed of contiguous odontodes, surrounds the older crown. Some scales appear transitional between the umbellate and Pultschuppe form, and others that have multiple upright pointed tubercles are probably branchial denticles (Fig 7M).

Thin sections show that the scales grew by appositional addition of odontodes laterally and posteriorly (Fig 7N and 7O); shorter, more upright odontodes are added anteriorly. The thin bone bases show several inner growth zones having separate bases, with bases underlying the whole scale formed only in the later growth periods (Fig 7N and 7O). Each odontode has a relatively wide pulp canal, surrounded and partly infilled by ‘Spiralfasern’, and interconnected by radial and circular canals. The ‘Spiralfasern’ structure (*sensu* Gross) [54], rather than being fibrous, appears to be formed by spiralling rings of atubular dentine which developed centrifugally to partly fill the wide pulp canals. Fine branching dentine tubules permeate the outer solid areas of the odontodes. Innermost (oldest) odontodes have separate? acellular bone bases, but the youngest growth zones have bases which underlie new and old growth zones. Sharpey’s fibres extended through the full-width zones, but not the individual odontode bases. Horizontal section of a Pultschuppe scale shows centrifugal growth zones (Fig 7P and 7Q).

### Cartilage

**Fig 8:**
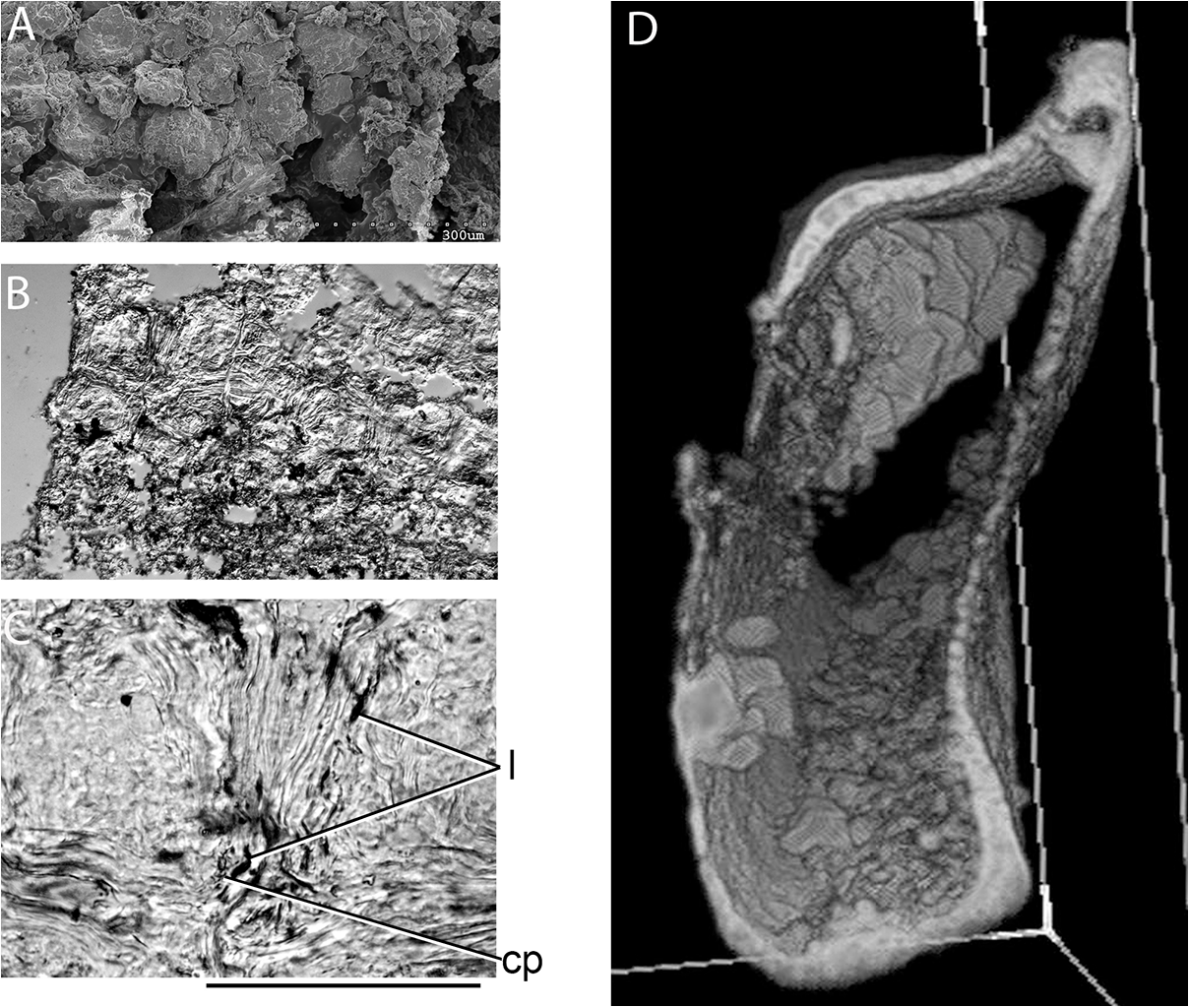
Structure of *Gogoselachus lynbeazleyae* endoskeleton. (A–C) *Gogoselachus* calcified cartilage (A) SEM WAM 09.6.145–031 showing tessellate layout (B, C) horizontal section through tissue WAM 09.6.145–032 (D) transverse CT scan of right Meckel's cartilage. Scale bar = 0.1 mm in (C). Abbreviations: cp, cell processes; l, lacunae.

**Fig 9:**
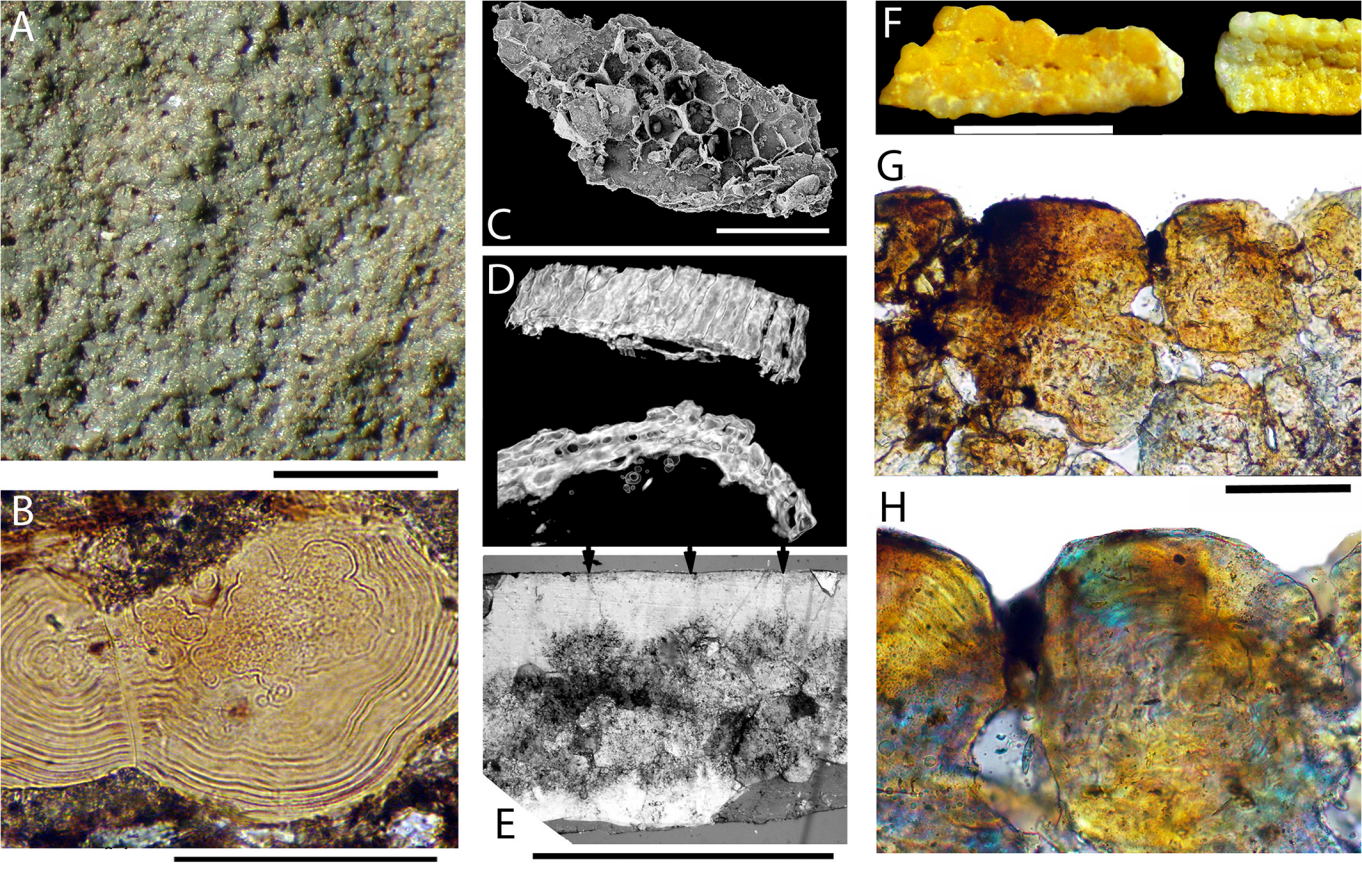
Comparative endoskeletal tissues in other Devonian gnathostomes. (A) globular (or granular) calcified cartilage preserved in the head region on stem chondrichthyan *Doliodus problematicus* NBMG 10127. (B) vertical thin section of large calcified cartilage globules in *Euthacanthus macnicoli* NMS G.2010.7.40. (C–E) placoderm endoskeletal tissues (C) SEM of QMF53545, perichondral bone sheet with honeycomb ‘cells’ in an antiarch from the upper Lower Devonian Cravens Peak Beds, western Queensland. (D) scans of perichondral bone from the Early Devonian *Parabuchanosteus murrumbidgeensis* from Taemas, ANU V24x. (E) section of sheet of crystalline calcite associated with a specimen of antiarch placoderm *Bothriolepis canadensis*; arrows show separation between individual crystals. (F–H) prismatic calcified cartilage of an undetermined shark from the Early Carboniferous Laurel Formation, northwestern Australia, QMF57586, QMF57587. (F) transmitted light image of QMF57588 showing variation in tesserae size (G, H) vertical section showing layout of tesserae, with prismatic structure visible under cross-nicols in (H). Scale bars = 1 mm in (A), (F), 0.1 mm in (B), (E), (G).

The endoskeletal elements are formed of mineralized tessellated cartilage (Fig 8) that varies in thickness, corresponding to the number of layers of tesserae. The Meckel’s cartilages, scapulocoracoids and the median ventral section of the basibranchial unit have two layers, whereas the gill-arch cartilages and the articulatory block on the scapulocoracoid are finer, with mostly one but rarely two layers. Thin sections of loose cartilage fragments (Fig 8B and 8C) show a structure similar to typical shark tessellated calcified cartilage, with subpolygonal tesserae showing rings and waves of Liesegang [55]. However, rather than having fibrous connections between the tesserae as found in modern sharks [56], the tesserae are mostly surrounded by a matrix incorporating elongate lacunae, some with fine processes extending from them (Fig 8C). CT scans (Fig 9H) show the varying thickness of the cartilage in different areas of the endoskeletal elements (Fig 8D).

## Discussion

Comparison of *Gogoselache lynnbeazleyae* with chondrichthyan taxa known from articulated specimens is hampered by the lack of a neurocranium and palatoquadrate, as many of the diagnostic characters recognized in other genera and families are components of these structures. Of the endoskeletal elements which are preserved, the Meckel's cartilages are characterised by having a sustentaculum, or retroarticular flange, but this is also found in other Palaeozoic sharks including xenacanthids and some cladoselachians, as well as modern *Ginglymostoma* (nurse sharks), in which the second division of the adductor muscle complex is inserted partly on its lateral rim, but may also extend further back to insert onto the articulation between the hyomandibula and ceratohyal [57–59]. Inspection of the articulated and very well preserved xenacanth figured by Hotton [57] shows that the sustentaculum does not project appreciably backwards, but merely overlies and covers the proximal end of the ceratohyal and the distal part of the hyomandibula. Part of the adductor musculature might have anchored on the sustentaculum in the Palaeozoic taxa. With regard to the jaw articulation surface, the orientation of the cotylus and the mandibular process on the same plane is comparable to the generalized condition seen in osteichthyans [33]. The dental trough shows no evidence of the scalloping present in many Palaeozoic sharks[51, 52]

A large fossa like that on the posterior end of the ceratohyal has been observed in some Paleozoic and Mesozoic sharks including hybodonts, xenacanths, and ctenacanths [57,60, 61,62] and also in some modern lamnid sharks. For example, it is very well developed in modern *Carcharias* [58] where it is empty with no ligament or muscle attachment (and is absent in *Squalus*, MIC, JAL pers. observ.), so there is no certainty which, if any, muscle or ligament attached in this pit in *Gogoselachus*. There is no pit in *Ozarcus* [21]. It has a very long, thin ceratohyal, with a proximal articular surface for the epihyal, and a distal one for the hypohyal. The ceratohyal is known in only a few fossil sharks; however, where we can see the morphology, there is often a huge pit at the posterior end (e.g., *Orthacanthus*, various ‘ctenacanths’ and hybodonts [22,57,61,62]). The shape of the ceratohyal has not been determined in Cleveland Shale *Cladoselache*. The 3-D ‘cladoselachian’from Kentucky, has a deep pit in the ceratohyal. It pinches the interior lumen so much that it has the articular end has the appearance of a separate posterior piece (originally interpreted as a separate interhyal by Maisey [44]). We conclude that the deep pit in the ceratohyal is more common in early sharks than previously recognised.

The uncompressed, original shape of the scapulocoracoid is known in few other fossil sharks. The Cretaceous hybodont *Tribodus limae* [63] and the Permian xenacanth *Orthacanthus texensis* [57] are similar to *Gogoselachus* in having a transverse posterior ridge for the fin basal articulation. The scapulocoracoid is much narrower and less angled than that of the slightly older Middle Devonian (Eifelian) *Antarctilamna prisca* (JAL, GCY pers. obs.). The presence of only two rather than three fin basal articulation facets on the scapulocoracoid is not unusual, as there is frequently no trace of a mesopterygial facet between the pro- and metapterygial facets in modern elasmobranchs and hybodonts with tribasal pectorals [63]. Overall the muscle attachments on the *Gogoselachus* scapulocoracoid demonstrate remarkable conformity with the myological pattern of recent chondrichthyans, including the chimaeroid *Hydrolagus*.

The morphology of the teeth is unique among the Devonian Chondrichthyes, showing a mix of features found in several shark groups. The crown is somewhat similar, mostly in the proportions of the cusps, to that of *Ctenacanthus concinnus* [64] and *Cladodoides wildungensis* [65,66], but the median cusps of the latter two are labially flattened whereas in *Gogoselache lynbeazleyae* they are biconvex. The asymmetry in Devonian ctenacanthids, albeit common, is never advanced to a point of occurrence of an intermediate cusplet only on one side. Moreover, the tooth base in ctenacanthids is provided with a distinct orolingual button and a straight, shelf-like basolabial projection, which are missing in *G*. *lynbeazleyae* teeth.

**Fig 10:**
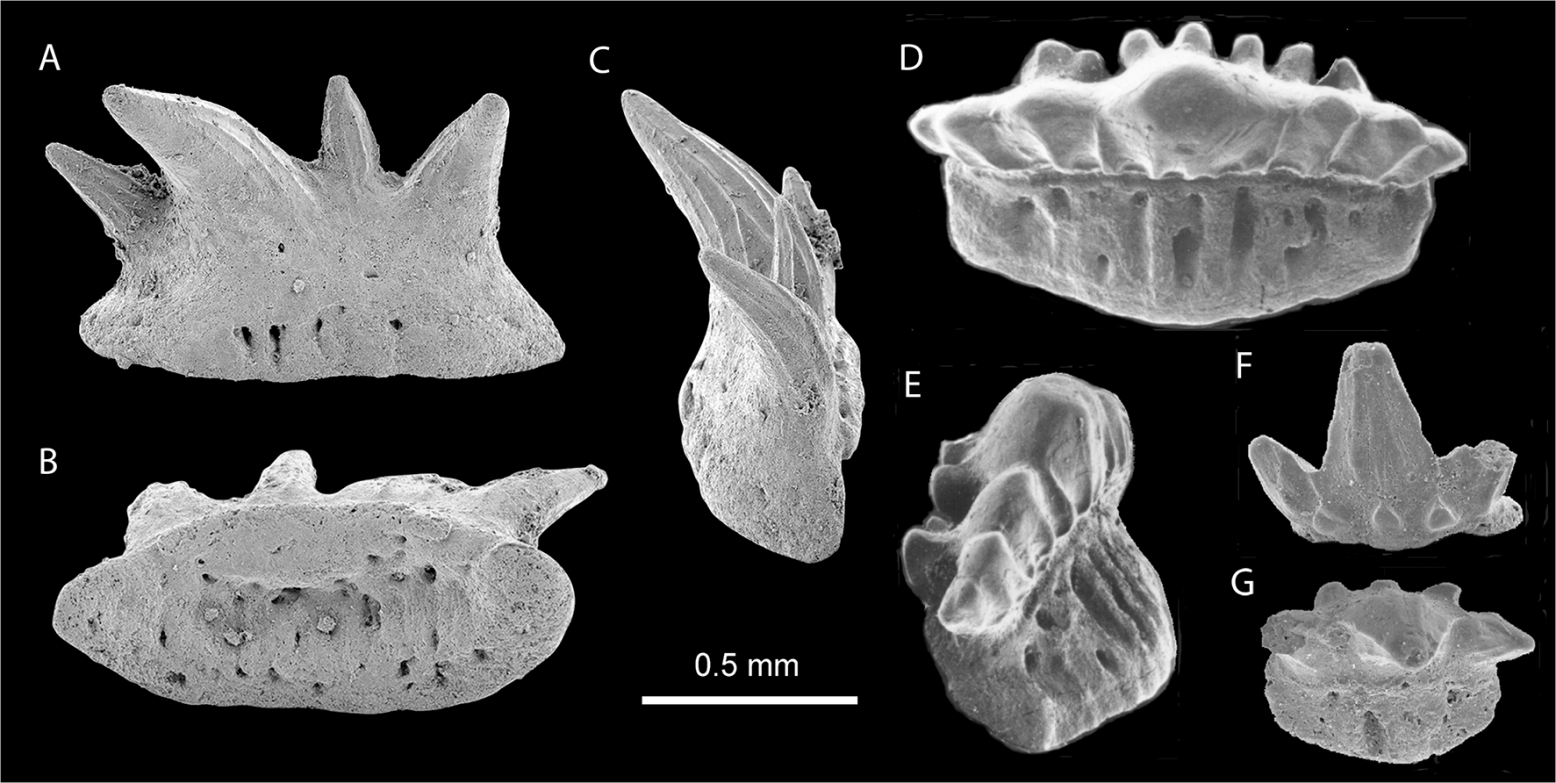
Teeth of sharks from the Upper Devonian of central Iran. (A–C) *Arduodens flammeus* Hairapetian & Ginter, 2009, from Chahriseh, AEU 610, in lingual, aboral, and lateral views. (D–G) *Deihim mansureae* Ginter, Hairapetian and Klug, 2002. (D, E) Holotype, IGPUW/Ps/5/1, from Hutk, in oral and lateral views. (F, G) Putative anterior tooth, AEU 239, from Hodjedk, in labial and oral views. Scale bar = 0.5 mm.

A recently described Iranian shark *Arduodens flammeus* [67] (Fig 10A–10C), from the upper Frasnian of Kale Sardar and the lower Famennian of Chahriseh, central Iran, has teeth similar to those of *G*. *lynbeazleyae*. The general form of crown and base of the two forms are very similar in both taxa, except the teeth of *Arduodens* do not have labial accessory cusplets, which are characteristic in *G*. *lynbeazleyae*. These cusplets are the most interesting character of the teeth in the new species. Only two other Devonian forms possess this feature, *Tamiobatis vetustus* sensu [22] and *Deihim mansureae* [45]. In *Tamiobatis*, the cusplets are very numerous (about 20), relatively short and needle-like, covering the whole basolabial region of the crown, unlike those in *Gogoselache lynbeazleyae*. The cusplets in *Deihim* (Fig 10D–10G) are distributed in a manner similar to that of *G*. *lynbeazleyae*. Unfortunately, all the specimens of *D*. *mansureae* come from a turbulent, shallow water environment of the Iranian Platform, so the teeth are usually highly abraded and only basal parts of the cusplets remained. Nevertheless, the shape of these damaged parts are comparable to the broken cusplets in *G*. *lynbeazleyae*, with the evident oral direction of their upper parts (compare Fig 5A and 5B).

Based on isolated teeth from the Famennian of Iran, Ginter et al. [45] proposed a strong heterodonty within the dentition of *D*. *mansureae*. Most of the specimens attributed to that species are various forms of crushing teeth. Only two specimens can be considered as clutching teeth, and these were tentatively included in *D*. *mansureae* because of the structure of the base and the presence of labial accessory cusplets. It was suggested [45] that the teeth were from the anterior tooth families and the whole dentition functioned in a way similar to modern *Heterodontus*, with a few minute anterior clutching teeth and a large lateral and posterolateral crushing apparatus ([68] fig24C). However, the reconstructed whole dentitions of *G*. *lynbeazleyae* and *D*. *mansureae* were completely different, with a total absence of crushing teeth associated with WAM 09.6.145. Therefore, we consider *G*. *lynbeazleyae* and *D*. *mansureae* to belong to separate, albeit probably related, genera.

A single tooth from the Upper Devonian of the Canning Basin, Western Australia, *Emerikodus ektrapelus* [69] bears some similarity to the new form. The preservation of the tooth is not very good, but the form of the main parts of the crown and base are quite similar to those of *G*. *lynbeazleyae*. However *Emerikodus* displays one very unusual feature: the row of accessory cusplets is apparently situated on the lingual, and not labial, side of the crown.

The presumed normal body scales of *Gogoselache lynbeazleyae* appear intermediate between the ‘cladodont’ type *sensu* Gross [53] and the ctenacanthid type *sensu* Reif [70]. The ‘cladodont’ scale category was based on isolated scales from the Middle Devonian Ohio bonebeds [54,71] and the Upper Devonian of Iowa, Rhineland and Harz [54]. The scales are characterized by having a base formed of lamellar bone layers penetrated by Sharpey's fibres, and a crown plate comprising circular or semicircular zones of odontodes; growth was by addition to the margin of the scale crown and a new (cellular or acellular) bone layer under the whole base.

The ctenacanthid type is based on scales of the articulated sharks "*Ctenacanthus* " *costellatus* Traquair and *Goodrichthys eskdalensis* Moy-Thomas from the Lower Carboniferous of Scotland, as well as isolated scales from Permian and Triassic deposits [52, 70]. The ctenacanthid type is characterized by hollow crowns ornamented anteriorly with strong ridges, neck canals restricted to the posterior, and a concave basal plate showing growth rings [70]—in effect, the separate bases of odontodes. Reif [70] noted that scales of the Upper Devonian “*Ctenacanthus*” cf. *C*. *clarki* are of the cladodont rather than ctenacanthid form. In *Gogoselache*, thin sections show that new basal layers underlying the whole scale are only added after the first two or three crown growth zones have formed, with the inner (paired) odontodes having individual bases. Thus ‘young’ scales could be classified as ctenacanthid type, while ‘older’ scales appear transitional between cladodont and ctenacanthid type. As noted by Williams for his type I scales of *Tamiobatis vetustus* sensu Williams [22] the crowns of *Gogoselache* normal body scales resemble those of isolated scales from the Middle Devonian bonebeds of Ohio for which Wells [71] erected the taxon *Cladolepis gunnelli*.

The *Gogoselache* scales include morphotypes comparable with the squamae umbellatae (edging sensory lines), tesserae coronatae (dorsal head), tesserae stellatae (ventral head), and Pultschuppen (?branchial) of the Late Silurian-Early Devonian acanthodian *Nostolepis striata* [53]. Like *N*. *striata*, normal body scales of *Gogoselache* show a wide variation in size and shape, but with appositional crown growth zones rather than the superpositional growth of *N*. *striata* (and most other acanthodians). *Gogoselache* has all scale forms I–IV that were illustrated by Williams ([22] Fig 6) for “*Tamiobatis vetustus”*. He noted that his type IV, stellate scales, are also “seen in profusion on other Cleveland Shale cladodont sharks” ([22] p.258). Unfortunately, histological structure of “*T*. *vetustus*” scales is not known, but their bases are comparable with those of *Gogoselache*, being concave and smooth except for scattered pore openings. Gross ([54] p.95) also consigned stellate platelets to his scale-based taxon *Maplemillia costata* Gross, 1973, from the Upper Devonian Maple Mill Shale, Iowa. Gross was uncertain whether the affinities of *Maplemillia* were with the Acanthodii or the ‘Cladodontida’. Odontodes on all *Gogoselache* scale varieties show the same distinctive histological structure as in body scales and stellate platelets of *M*. *costata*, with a spiralling structure surrounding the pulp canals. Gross ([54] p.93) interpreted the spiralling lines as fibres, ‘Spiralfasern’. However, rather than being fibres, these lines represent spiralling rings of atubular dentine which partly filled the pulp canal centrifugally. This type of structure is also found in *Cladolepis gunnelli* and Late Palaeozoic elasmobranchs [54].

Several occurrences of ctenacanthid and cladodont type scales have been reported from eastern and western Australian Middle to Late Devonian microvertebrate assemblages [72–74]. Other rich assemblages from the? upper Givetian Aztec Siltstone at Mt Crean, Antarctica include ctenacanthid-type scales similar to those of *Gogoselache*, that are most likely from *Antarctilamna prisca* [75]. The acanthodian and chondrichthyan microremains in the Mt Crean assemblage [76] show a marked similarity to early Frasnian faunas from Iran [77].

Comparing *Gogoselache* scales with those of older taxa, the flank scales with their concave bases and polyodontode crowns resemble those of *Leonodus* Mader from the Lochkovian of northern Spain [78]. *Doliodus problematicus* from the early Emsian of New Brunswick, Canada [15], and *Antarctilamna prisca* Young from the Givetian of Antarctica [75]. Unlike *Gogoselache*, all of these taxa have diplodont teeth, indicating that the scale form is probably ancestral to both shark lineages determined by tooth morphology.

The structure of the calcified cartilage in the endoskeleton of *Gogoselache* is of interest as an apparent transitional form between GCC and PCC. CT scans and thin sections show a calcified tissue forming the outer shell of endoskeletal elements, more derived than the simple calcified cartilage comprising separate globules found in osteostracan agnathans [55], acanthodians [79] and some stem chondrichthyans (Fig 9A and 9B). Placoderms differ from other gnathostomes in having perichondral bone plus globular (GCC) and/or uncalcified cartilage forming their endoskeleton (Fig 9C and 9D) in instances where such elements are preserved at all [53,73,80].

GCC and PCC both have rings and waves of Liesegang, but PCC differs from GCC in being birefringent, and comprising one or more tessellated layers of polygonal tesserae connected by collagen fibres [54]. It is perhaps relevant to the development of prisms in calcified cartilage, that one form of crystalline calcite has a layer of crystals of similar size and optic properties as the prismatic tesserae in PCC (Fig 9E; specimen mistakenly labelled as calcified cartilage in [81]). The earliest record of tessellated calcified cartilage (PCC), a tissue considered a diagnostic character of chondrichthyans (Fig 9F–9H), is from the? upper Lower Devonian Cravens Peak Beds, northwestern Queensland], and is presumed to derive from *Mcmurdodus whitei* [82]. The histology of tessellated calcified cartilage in articulated sharks from the Late Devonian, however, has been little studied. Most previously described articulated sharks of Late Devonian age are from shale deposits, with poor potential for preservation of histological details.

We sectioned tessellated calcified cartilage (Fig 9F–9H) from the Early Carboniferous Laurel Formation, northwestern Western Australia, which proved to be typical PCC: vertical sections of the layered tesserae show birefringence, and there is no cellular matrix between the tesserae. In modern sharks, only the outer region of the directly subperichondral tesserae is PCC, with the inner region and subsurface tesserae being GCC [75]. The tesserae are held together by unmineralized fibres; at least in some taxa, chondrocytes also occur in the intertesseral joints [83], but no incidences have been recorded of osteocyte-type cellular processes, as are found in *Gogoselache*. The mineralized tissue structure preserved in *Gogoselachus lynbeazleyae* is thus far unique. Multiple-layered tesserae are well known in all parts of the endoskeleton in ctenacanth and xenacanth chondrichthyans, even in regions presumably not subjected to high stresses [83]. Multiple layering is only found in modern chondrichthyans when thickening develops around high stress areas, and internal struts of tesserate calcified cartilage (also in high-stress areas) in the jaw cartilages [83]; similar struts are found in the jaws of *Tribodus* [62]. Hybodonts, like modern sharks/rays, typically only have a single layer of tesserae, a condition also seen in Devonian chondrichthyans such as *Pucapampella* and *Cladodoides*.

## Conclusions

*Gogoselachus lynbeazleyae* is an exceptionally preserved acid-prepared fossil chondrichthyan from the late Gogo Formation Devonian of Australia. Its lower jaws have an expanded cotylus and small mandibular knob, and the slender scapulocoracoid has just 2 facets for radial articulations. The teeth have well-developed labial cusplets and their structure is intermediate between the ctenacanthiform and symmoriiform condition. The distinctive calcified cartilage forming the endoskeleton has multiple layers of nonprismatic subpolygonal tesserae separated by a cellular matrix. This suggests *Gogoselachus* represents a transitional step toward the tessellated prismatic calcified cartilage which is today recognized as the main diagnostic character of the Chondrichthyes.
