# Correction: First Shark from the Late Devonian (Frasnian) Gogo Formation, Western Australia Sheds New Light on the Development of Tessellated Calcified Cartilage

**DOI:** 10.1371/journal.pone.0131502

**Published:** 2015-06-22

**Authors:** John A. Long, Carole J. Burrow, Michal Ginter, John G. Maisey, Kate M. Trinajstic, Michael I. Coates, Gavin C. Young, Tim J. Senden

The affiliation for the third author is incorrect. Michal Ginter is not affiliated with the Paleontology Section but with the Faculty of Geology, University of Warsaw, Warsaw, Poland.

## References

[1] Long JA, Burrow CJ, Ginter M, Maisey JG, Trinajstic KM, Coates MI, et al (2015) First Shark from the Late Devonian (Frasnian) Gogo Formation, Western Australia Sheds New Light on the Development of Tessellated Calcified Cartilage. PLoS ONE 10(5): e0126066 doi: 10.1371/journal.pone.0126066 26020788 10.1371/journal.pone.0126066 PMC4447464

